# Neuropsychobiological transdiagnostic risk mechanisms for aggression in mental disorders across the life span

**DOI:** 10.1186/s12888-026-08359-5

**Published:** 2026-09-30

**Authors:** Ute Habel, Andreas Reif, Sabine Herpertz, Kerstin Konrad, Christine Freitag, Tobias Banaschewski, Thomas Frodl, Catherine Barnes-Scheufler, Lisa Berg, Katja Bertsch, Natalya Chechko, Andreas G. Chiocchetti, Christine Ecker, Gabriele Ende, Antonia Fritsch, Chiara Gramegna, Michael Hanke, Nathalie Holz, Philippa Hüpen, Ina Kuschel, Klaus Mathiak, Annika Maas, Andreas Meyer-Lindenberg, Corinne Neukel, Christian Paret, Michael Plichta, Vanessa Reindl, Wolfgang Retz, Jaqueline Scharf, Christian Schmahl, Henry Schirok, Heike Tost, Daniel Turner, Oliver Tüscher, Lisa Wagels, Carmen Weidler, Jana Zweerings

**Affiliations:** 1 Institute for Translational Neuroscience and Clinical Psychology, Uniklinik RWTH, Aachen, Aachen, Germany; 2 INM-10, Research Center Jülich, Jülich, Germany; 3 Department of Psychiatry, Psychosomatic Medicine and Psychotherapy, University Hospital Frankfurt, Goethe University, Frankfurt, Germany; 4 Department of Psychiatry and Psychotherapy, University of Heidelberg, Heidelberg, Germany; 5 Institute of Medical Psychology and Medical Sociology, Uniklinik RWTH, Aachen, Aachen, Germany; 6 Department of Child and Adolescent Psychiatry, Psychosomatic Medicine and Psychotherapy, University Hospital Frankfurt, Goethe University, Frankfurt, Germany; 7 Central Institute of Mental Health, Medical Faculty Mannheim, University of Heidelberg, Mannheim, Germany; 8 Department of Psychiatry, Psychotherapy and Psychosomatics, Uniklinik RWTH Aachen, Aachen, Germany; 9 Department of Psychology I (Biological Psychology, Clinical Psychology, and Psychotherapy), University of Würzburg, Würzburg, Germany; 10 INM-7, Research Center Jülich, Jülich, Germany; 11 Institute of Systems Neuroscience, Medical Faculty, Heinrich-Heine-University, Düsseldorf, Germany; 12 Institute for Forensic Psychology and Psychiatry, Saarland University, Homburg, Germany; 13 Leibniz Institute for Resilience Research (LIR), University Medical Center Mainz, Mainz, Germany; 14 Department of Psychiatry, Psychotherapy and Psychosomatic Medicine, University Medicine Halle (Saale) of the Martin Luther University Halle-Wittenberg (MLU) and German Center for Mental Health (DZPG), Halle (Saale), Germany

**Keywords:** Aggression, Mental disorders, Negative valence system, Cognitive system

## Abstract

**Background:**

Aggression in mental disorders (AMD) is a prevalent symptom, poses difficulties to the clinical setting and has far-reaching societal implications. The biological basis of different manifestations, developmental courses, and disease-specific mechanisms remains largely unknown. The lack of understanding of the complex mechanisms underlying AMD due to the heterogeneity of aggression and psychiatric disorders themselves, will be addressed by studying a longitudinal transdiagnostic cohort of patients suffering from mental disorders dimensionally quantified with respect to aggressive behavior.

**Methods:**

Capitalizing on the Research Domain Criteria “negative valence system” (NVS) and “cognitive system” (CS), multilevel measurements of behavioral and biological systems provide a harmonized dataset at three study sites (Frankfurt, Mannheim/Heidelberg, Aachen; 900 adults, 200 children/youth) including forensic satellites (*n* = 150). A cross-sectional matched control group (300 adults, 100 youth) will also be included.

**Discussion:**

The goal of the study is to provide a multilevel characterization of patients, assessing dimensional phenotypic measures across time points and diagnoses, to finally define aggressive biosignatures.

**Trial Registration:**

The present study has been registered at https://drks.de/register/en/trial/DRKS00035731 (number of registration: DRKS00035731; date of registration: 20/02/2025).

## Background

Present in all species, aggression is an evolutionarily preserved behavior which includes harming another living being to protect, gain, or defend something of value. As a broad and commonly used definition, aggression is a “behavior that is intended to harm another person who does not wish to be harmed” (e.g. [1]). Aggression is a multifactorial phenomenon, explained by complex interactions of various biological, psychological, cognitive, and environmental factors that determine and contribute to situational aggressive behavior. Complex neurocognitive and neurobiological pathways are assumed to mediate multiple manifestations and thereby define aggressive biosignatures. Importantly, many mental disorders (whether developed in childhood or adulthood) are associated with an increased likelihood of aggression [2]. In the clinical context, aggression is prominent as a symptom of mental disorders [3], including, for instance, schizophrenia, borderline personality disorder (BPD) [4–6], autism spectrum disorder (ASD) [7], depression, attention deficit/hyperactivity disorder (ADHD) [8], substance abuse and dependence, as well as bipolar disorder, and is especially prominent in forensic patients [9–11], in conduct disorder (CD) [12], and antisocial personality disorder (ASPD) [13]. In addition to the negative social, socio-economic, and personal consequences, aggressive behavior in patients worsens their treatment outcome [14]. Despite the clinical relevance of AMD, there is a lack of understanding the neurobiological substrates and mechanisms that underlie different types of aggression in mental disorders. As a consequence, the relevant pharmaceutical and psychotherapeutic interventions are both insufficient and inefficient, especially as aggression is not often specifically targeted per se. So far, research on the nature, origin, and stability of AMD has often focused on individual disorders or isolated aspects of aggression, which is insufficient for identifying transdiagnostic facets and may bias the understanding of aggressive behavior elicited by specific disease symptoms. Research in the field of aggression in mental disorders (AMD) faces critical challenges. These include the need for comprehensive clinical characterization, understanding developmental trajectories and the multifactorial etiology of AMD.

A comprehensive model of aggression should address the (neuro)biological substrates of AMD in association with environmental, developmental, and cognitive-emotional aspects. Dysfunctions in the cognitive- and emotion- processing systems are a hallmark of mental disorders. Hence, the question is, which subprocesses are involved in, affected by, and contribute to aggression? The scientific consensus is that, to a large extent, it is the processing of threat, frustration, and cognitive control that determines aggressive behavior [15–18]. The National Institute of Mental Health (NIMH) has proposed related constructs within the RDoC framework (i.e., the “acute threat/potential harm” and “frustrative-non-reward” constructs of NVS and the cognitive control construct of the CS). The RDoC perspective allows the consideration of these domains individually, as well as being intertwined, advocating investigations of imbalances and dysfunctions of different neurobiological systems. Major endophenotypes under investigation in this planned study include genetic, environmental, neuropsychological, neuroimaging and hormonal signatures that all need to be assessed with respect to their role for aggression to translate findings from the molecular to the systems level and, finally, the behavioral phenotype.

The application of functional neuroimaging, which has demonstrated the importance of NVS and CS for aggression in patient groups, proposes an imbalance of attenuated prefrontal top-down control (reflective of the CS) and excessive bottom-up activity of the limbic and reward regions in impulsive or reactive aggression (reflecting the NVS, e.g. [19, 20]). Consequently, these regions are active during complex social interaction paradigms involving provocation and frustration on the one hand and emotion regulation and behavioral inhibition on the other [20–23]. Heightened sensitivity to threat-related cues, or misinterpretation of neutral cues as threatening, may over-activate the acute threat system, which may elicit anger or fear and a potentially aggressive response if cognitive control regions cannot counteract, as shown for BPD [24]. In BPD and CD, the fronto-limbic networks involved in emotion processing and the frontal regions involved in regulation have shown abnormalities in structure and function [5, 8, 25–27]. Similarly, impairments in emotion processing and regulation networks contribute to enhanced aggressiveness in ADHD, ASD, and disruptive behavior disorders [28, 29]. In children and adolescents with CD, altered fronto-limbic regulation has been observed [30, 31], similar to individuals with high trait aggression [32]. We, along with other researchers, have previously shown that a complex interplay of different factors such as genetics, hormones, and neurotransmitter systems together with environmental influences contributes to the development of aggression. Heritability of aggressiveness has been estimated at 50–60% [33], and it is even higher in individuals with severe aggressive behavior and high levels of callous-unemotional traits [34]. To date, most studies have been “traditional” candidate gene association studies, focusing on the serotonergic and dopaminergic systems, as well as genes related to stress hormones or the oxytocin system [22, 33, 35, 36]. These however fall short, as around 50% of the variance in aggressive behavior may be explained by complex, polygenetic influences [33, 34], and only the sum of genetic variance in numerous genes (typically comprising thousands of polymorphisms) predicts a meaningful share of heritability. This sum is operationalized by polygenic risk scores (PRS) derived from genome-wide association studies (GWAS). Most genetic variations, which increase the liability of aggressive behavior, interact with biological or psychosocial environmental risk factors [37]. A GWAS analyzing data of over 700 violent offenders [38] strongly suggests complex interactions with childhood stress, trauma, gender, and hormones - all of which will be included in our study (see also [39–41]). While there is a paucity of specific, hypothesis-free GWAS on aggression with sufficient sample sizes, a recent synthesis of GWAS with biosignatures related to aggressive behavior has suggested RBFOX1, an RNA binding protein that affects splicing and mRNA stability of thousands of genes, as a promising candidate [42]. This finding has been validated and replicated [43]. A promising approach is to study PRS together with environmental risk and neurobehavioral phenotyping.

There is furthermore a clear gender bias in aggressive behavior at large, but also the fact that aggressive behavior in females is often overlooked, probably since women seem to predominantly display verbal and indirect aggressive behavior [44]. Meta-analytic and qualitative reviews on gender differences show greater aggression in males than in females with most prominent differences in direct, physical aggression across different contexts [45–47]. This consistent gender difference seems to disappear under high and/or prolonged provocation settings [23]. Evidence points towards complex sex-specific gene-by-environment interactions in aggression [48]. There is only minor evidence for gender differences with regard to the respective neurobiological underpinnings of aggression (e.g. [21, 49]), due to a paucity of studies explicitly addressing such differences, but rather relying on men only when studying brain and hormonal mechanisms. Aggression, in the context of mental disorders or outside of it, is found much more often in males than in females. However, relative risk for offenses can be even higher in female patients compared to controls due to the low risk in healthy females [50]. Hence, AMD is also present in females and may often be overlooked due to normative social assumptions. Additionally, the ultimate and proximal causes for aggression might be quite different in females.

Furthermore, female and male sex hormones play a role in aggression, with increased testosterone concentrations observed in female and male BPD patients [51]. Testosterone may play a significant role in the neuromodulation of aggression [40, 52]. It may interact with contextual changes as well as genetic influences [22, 36, 41, 53], affecting behavior in challenging and threatening situations [54–56] by influencing key brain regions for social threat processing such as the amygdala, the hypothalamus, and the periaqueductal gray (PAG) [22, 41, 57–60], suggesting an involvement in the NVS. While reduced estrogen has been found in females with CD [40], and cortisol awakening responses were positively related to anger and aggressiveness in female patients with BPD [51], the role of female-specific sex hormones in aggressive behavior has not yet been studied in detail.

In addition to the biological factors, psychological and psychosocial consequences of negative environmental exposures also comprise impairments in social cognitive and affective processing [61] and may therefore enhance the risk for aggression in patients. Adverse Childhood Experiences (ACE), including abuse and neglect as well as early exposures to urban environments, have been identified as important risk factors for aggressive behavior and psychopathology (e.g. [28, 61–63]). The type and timing of ACE are of particular interest in terms of prevention and treatment [64]. During certain vulnerable developmental phases, the risk of subsequent ACE-related disorders seems to increase [65, 66].

The present initiative pursues the approach to characterize, for the first time, specific patterns of aggression and their emergence in mental disorders from the perspective of dysfunctions or imbalances within these systems. In addition to shedding light on the complexity of aggression, it is critically important to specify how aggressive behavioral patterns stabilize throughout the development and course of the disease. Our initiative pursues a multidimensional, developmental, and longitudinal approach to advance understanding of the neurobiological mechanisms and associated neuropsychological processes in interaction with short- and longstanding environmental influences and clinical features. We aim to provide a harmonized recruitment approach across sites and disorders to dimensionally assess aggressive biosignatures and their developmental trajectories over the lifespan. This is essential in order to overcome these disease-oriented assessments commonly carried out in research as well as in clinical practice.

**Fig. 1:**
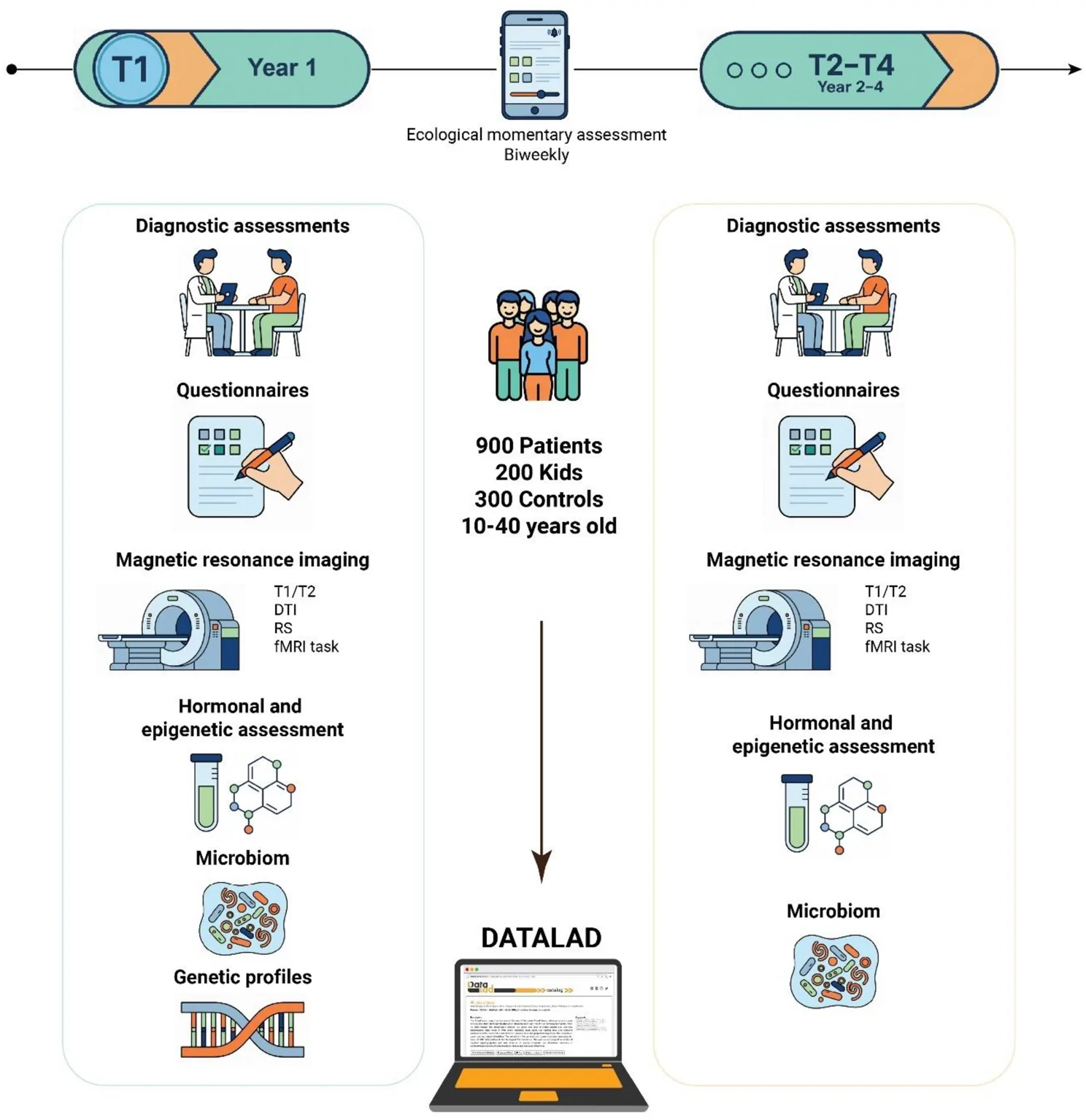
Design of the longitudinal study. The figure displays the measurements and variables at the different time points of the first project funding phase

With that we pursue the scientific aims of our newly established collaborative research center on the “Neuropsychobiology of Aggression: a transdiagnostic approach in mental disorders” (funded by the German Research Foundation, DFG: SFB/TRR 379) by a) providing transdiagnostic characterization of AMD including the assessment of dysfunctions in cognitive control (CS) and threat/potential harm and frustrative-non reward (NVS), by examining shared and disorder-specific mechanisms of aggressive behavior and; b) specifying neurobiological mechanisms on the pathway to aggression, to advance our understanding of neurocognitive and neurobiological mechanisms and situational/environmental influences determining specific behavioral patterns of AMD. Here, we define these specific patterns and the underlying mechanisms as biosignatures of aggression. We specifically hypothesize that based on the NVS/CS characterization (questionnaires, see Table 1), and genetic, hormonal (testosterone, estradiol, oxytocin, cortisol) and brain correlates (structural markers, diffusion tensor imaging (DTI), resting state fMRI, active resting state fMRI during an aggressive movie), we will identify up to six different clusters (**hypothesis 1**, see Figure 1) representing different aggression biosignatures across diagnostic categories. These biosignatures are related to psychopathological symptom clusters (assessed with diagnostic and clinical assessments and self-assessments see Table 1), reveal environmental influences (assessed with EMA, **hypothesis 2**), can vary over time and show a dependence on psychopathological symptom clusters and functioning of the CS/NVS (**hypothesis 3**).

**Table 1:** Questionnaires and diagnostic assessments. Constructs that are measures are mentioned in the first column

	Adults		Children/youth	
	Instrument	Abbr.	Instrument	Abbr.
**Diagnoses**	Mini International Neuropsychiatric Interview	MINI-DIPS*	Diagnostic Interview in children and adolescents (German version)	Children-DIPS
	International Personality Disorder Examination for DSM-IV/ICD-10 - Screening	IPDE-Screening		
	International Personality Disorder Examination for DSM-IV/ICD-10	IPDE		
	DZPG - Minimum Data Set Core	MDS core*		
	Medical History Form	MHF	Medical History Form	MHF
**Bullying**			Olweus Bullying Questionnaire - Revised	OBQ-R
**Pubertal Status**			Pubertal Development Scale	PDS
**Peers Neighborhood**			SAHA peers and neighborhood	SAHA-P
**Stressful Life Events/ACE**	Childhood Trauma Questionnaire	CTQ	Childhood Trauma Questionnaire (Youth > 11 years old)	CTQ
**Social Network**	Social Support Questionnaire (Fragebogen zur Sozialen Unterstützung) K-14 Version	SSQ (FSozU)*	Social Support Questionnaire (Fragebogen zur Sozialen Unterstützung) K-14 Version	SSQ (FSozU)
**Constructs**				
**Aggression**	Reactive and Proactive Aggression Questionnaire	RPQ*	Reactive and Proactive Aggression Questionnaire	RPQ
	Modified Overt Aggression Scale	OAS-M*	Modified Overt Aggression Scale	OAS-M
	State-Trait-Anger-Expression-Inventory-II	STAXI −2*	State-Trait-Anger-Expression-Inventory-II Child and Adolescent	STAXI-2-KJ
	Life History of Aggression Scale	LHA		
**Callous-Unemotional Traits**	Inventory of Callous-un-emotional traits	ICU	Inventory of Callous-un-emotional traits (parents, youth)	ICU*
**Irritability**			Affective Reactivity Index	ARI
**CS: Impulsivity**	Barratt Impulsiveness Scale – Short Version	BIS-15*	Barratt Impulsiveness Scale – Short Version	BIS-15
**CS: Self-Regulation**	Difficulties in Emotion Regulation Scale	DERS-36*		
	Emotion Regulation Questionnaire	ERQ	Emotion Regulation Questionnaire for Children and Adolescents (Youth > 9 years old)	ERQ-CA
	Anger Rumination Scale	ARS*		
**NVS: Threat Sensitivity**	Questionnaire on Threat Sensitivity	TSS*		
	Behavioral Inhibition Scale	BIS/BAS	Behavioral Inhibition Scale	BIS/BAS
**NVS: Frustrative Non-Reward**	Questionnaire of Daily Frustrations	QDF*		
**Intelligence**	Wechsler Adult Intelligence Scale IV (subtest: Digit Span)	WAIS-IV-DS*	Wechsler Intelligence Scale for Children 5 (subtest: Digit Span)	WISC-5-DS
	Wechsler Adult Intelligence Scale IV (subtest: Vocabulary)	WAIS-IV – V	Wechsler Intelligence Scale for Children 5 (subtest: Vocabulary)	WISC-5-V
	Intelligence Screening	mini-q*		
			Wechsler Intelligence Scale for Children 5 (subtest: Matrix Reasoning)	WISC-5-MR
**Functioning**			Social and Occupational Functioning Assessment Scale (Youth > 11 years old)	SOFAS
			Children’s Global Assessment Scale	CGAS
**Psychopathology**	Adult Self Report	ASR*	Child Behavior Checklist Revised (parent) Youth Self Report (youth)	CBCL-R YSR
	Adult Behavior Checklist	ABCL*		
	Personality Inventory for DSM-5	PID-5-BF+M		
	ICD-11 Short Version			
	Level of personality functioning scale (brief version)	LPFS-BF*	Revised Child Anxiety and Depression Scale	RCADS
	Psychopathological assessment	AMDP*		
			Diagnostic System for Mental Disorders in Children and Adolescents based on the ICD- 10 and DSM-5 (DISYPS-III), parents form ADHD	DISYPS-KJ-III: FBB-ADHS
			Diagnostic System for Mental Disorders in Children and Adolescents based on the ICD- 10 and DSM-5 (DISYPS-III), parents form ODD	DISYPS-KJ-III: FBB-SSV
**Ecological Validity**	Ecological Momentary Assessment	EMA	Ecological Momentary Assessment	EMA

*Note.* * Instruments in bold will be assessed at all time points

The development of aggressive behavior that typically occurs with a higher frequency in boys than in girls from childhood to adulthood is part of normal personality development [67]. Over the course of development into adulthood, there is a general decrease in overt, impulsive, and reactive forms of aggression [68], and an increasing influence of genetic predisposition [33]. In case of childhood adversities and other bio-social risk factors, the age-adequate control of aggression is altered, leading to maladaptive aggressive behavior with clinical relevance [69]. In a normative sample from mid-childhood to early adolescence four trajectory groups of reactive aggression emerged: high stable aggression (7.6%), moderate decreasing (25%), low increasing (8.3%), and low stable aggression (59%) [70]. The longitudinal development of aggression and its endophenotypes, especially during the transition period from childhood to adulthood, is still poorly understood. Since most clinical studies have focused on the development of specific mental disorders associated with aggression, there is a paucity of studies using a truly transdiagnostic approach and considering relevant cognitive-emotional mechanisms in their causal relationship to the emergence of aggressive behavior. A further aim with this study is hence to identify sex-specific developmental trajectories in adolescents and young adulthood over critical transition periods and sex-specific patterns of dysfunctions in adults. The sample that we assess will be followed up annually longitudinally over the funding period of four years and the expected further phases of eight years. With this data we will be able to predict risk and resilience in relation to aggressive behavior and to assess the stability of AMD and (dys)function of CS and NVS over time, and in correlation with fluctuating psychopathological symptoms.

We here assume that developmental sex-specific differences emerge in the assessed dimensions (hormones, questionnaire, psychopathological assessments, neuroimaging markers) that are more pronounced in certain aggression biosignatures (**hypothesis 4**) and that the aggression biosignatures and their interrelatedness with psychopathological symptom clusters have predictive power for long-term development of aggressive behavior, also in forensic patients (**hypothesis 5**). Sex differences related to aggression biosignatures (in the variables mentioned above) will persist over long-term development (will be stable across measurement time points; **hypothesis 6**).

Taken together, understanding the complex nature of aggressive behaviors needs a multifactorial model that considers the role of neuropsychobiological and social factors. It is imperative that the mechanisms underlying aggression are simultaneously investigated on multiple levels in the same individual longitudinally, and in multiple models, for a complete understanding of the phenomenon, while studies in isolation will fail to provide a complete understanding.

## Methods

This study protocol complies with all relevant ethical regulations, and the review board of the Medical Faculty RWTH Aachen University Hospital approved the study (EK 24–270); the same applies to the review boards of Frankfurt Medical Faculty and Heidelberg Medical Faculty as well as Mannheim Central Institute of Mental Health, the participating recruitment sites. All participants will give oral and written informed consent and will receive financial compensation. For minors under 18, parents will give oral and written informed consent.

In this project, we will recruit and systematically phenotype in- and outpatients suffering from mental disorders that increase the predisposition towards aggressive behavior, covering the age span from 10 to 40 years upon study intake, and will follow them over the next 3.5 years. No randomization is required here in this within-subject design with annual repeated measures (planned for 12 years, funding is guaranteed for 3.75 years and has the option for two further renewals). The age range is based on evidence that aggression shows a peak in prevalence in young adulthood and a decrease across the life span but seems to be similarly prevalent across almost all age groups when examined among those who engaged in at least one violent act in the past year [71]. We will set up a prospective-longitudinal observational study including all three sites quantifying the degree of aggressive behavior. The contributing clinical departments are Departments of Psychiatry, Psychotherapy and Psychosomatics as well as Child and Adolescent Psychiatry, Psychosomatics and Psychotherapy at University Hospitals Aachen, Frankfurt, Mannheim (ZI) and Heidelberg. A smaller sample of patients from forensic hospitals in Duren and Viersen, the Hospital for Forensic Psychiatry Nette-Gut, Weißenthurm and Department for Forensic Psychiatry Rheinhessen, Fachklinik Alzey, as well as the Hospital for Forensic Psychiatry Pfalzklinikum Klingenmunster will also be included.

Exclusion criteria include dementia, severe mental retardation (IQ < 50), insufficient ability to read and write German (adults / parents or caregivers), inability to comply with the study protocol. Participants with severe somatic or neurological disorders that affect the central nervous system or hormonal functions (such as encephalitis, brain and hormone secreting tumors, dementia, epilepsy, fetal alcohol syndrome) will also be excluded. MRI contraindications will be further exclusion criteria. For healthy controls, any mental disorder according to DSM-5/ICDF0–ICDF4 is an exclusion criterion.

Patients will be initially screened and then asked for informed consent to the study after full explanation of the study protocol. For the baseline, diagnostic assessments will take place and in- and exclusion criteria will be checked. Categorical axis I and II diagnostics will be established using structured clinical interviews (MINI [72]; IPDE [73]). Data collection further includes sociodemographic information, self- and clinician-rating scales on psychopathology and aggression, experience sampling measurements using Ecological momentary assessments (EMA), current and retrospective analysis of electronic health records regarding conflict, aggressive acts and irritability; intelligence estimation, structural and functional brain measures (Anatomy: T1, T2, Diffusion Tensor Imaging: DTI, Functional imaging: fMRI (active) resting state) as well as hormonal, microbiome and genetic profiles. The sociodemographic information will be aligned with the DZPG (German Center for Mental Health) core dataset as will be RDoC relevant items e.g. on anger/aggression. Psychopathological assessment and questionnaires, epigenetic and hormonal samples as well as brain measures will be gathered annually. For a full schedule of visits and measures, please see Fig. 1. The study will permanently recruit patients suffering from a mental disorder, treated at one of the three hospitals, and follow them during the funding period.

Measurements include the following domains (see Table 1; Fig. 1):

1. Measures of and proxies for aggression (interview, clinical rating and self-report) to establish a transdiagnostic baseline prevalence estimation for AMD and to then describe the long-term trajectory of aggressive behavior.
2. The role of the RDoC NVS (e.g., subjective stress, anger, threat bias, affect states, daily frustrations) and CS systems (self-regulation, impulsivity) in persisting and desisting aggressive behavior will be measured using behavioral assessments and questionnaires.
3. Ecological Momentary Assessment (EMA) twice a week in a randomized sparse sampling method, and in boosting intervals following the MR measurements for two weeks with five prompts a day.
4. Hormonal, epigenetic, and environmental factors will be assessed annually. In addition, in adolescents, the pubertal development will be assessed with the Pubertal Development Scale (PDS) every year.
5. Brain structure and function: T1, T2, DTI, fMRI resting-state EPI sequence, aggression perception fMRI task. Where feasible, MR measures will be collected annually, and at a minimum every two years.
6. DNA will be collected at baseline or during study to store it in the biorepository for genome-wide analysis.
7. Microbiome.

Forensic patients will be assessed using questionnaire data as well as hormonal, epigenetic, and genetic data (whenever possible).

### Questionnaires:

For adults and children/adolescents we will measure the relevant constructs and functions with equivalent or age-adopted self-reported (or parent reported) questionnaires (see Table 1), and clinical rating scales wherever possible at baseline and every 12 months.

**EMA (ecological momentary assessment):** EMA measures will be obtained by self-ratings using a smartphone-app, based on a graphic user interface (JTrack). The EMA protocol will be developed and run through the JTrack platform. It is a secure, reliable and extendable open-source solution for remote monitoring in daily-life and digital-phenotyping. JTrack is an open-source (released under open-source Apache 2.0 licenses) platform for remote assessment of digital biomarkers (DB) in neurological, psychiatric and other indications. JTrack is developed and maintained at the Forschungszentrum Julich to comply with security, privacy and the General Data Protection Regulation (GDPR) requirements. JTrack also provides an online study management dashboard to monitor data collection across studies. To facilitate scaling, reproducibility, data management, and sharing, we integrated DataLad as a data management infrastructure.

We will use diaries to assess the number of conflicts and aggressive outbursts every four days continuously over the funding period.

For the momentary assessment of aggression, we will use the Aggression-ES-A [74] and additionally we include an item from the Aggression-ES [75] on physical attacks (see above). In the diaries, participants will be asked how many times they showed each behavior in the days since the last diary, and additional questions will capture the context, such as where they were and what they were doing at the time.

EMA holds significant potential to capture day-to-day dynamics of aggression. It also allows to capture momentary influences, which may include provocations, angry or hostile affective states, perceived stress, and intoxication. Therefore, EMA measures will also be assessed intensively in bursts following the aggression film perception and MR measurement for two weeks with regular prompts (5/day). These will capture provocations, negative affective states, anger, substance use, subjective stress, and the context (main activity, social and physical context) a person was in right before the prompt. For children, to avoid interference with school regulations, the number of intensive bursts will be reduced to fewer instances during the evening on weekdays, whereas there will be more bursts during the weekend.

**Hormones**: Saliva samples to measure testosterone (T), cortisol (C), estrogen (E), oxytocin (OT), and progesterone (PR) levels utilizing a highly standardized procedure (fixed time / day and in relation to menstrual cycle) will be collected. We will measure baseline levels for hormones annually (especially T, E, PR, C (awakening cortisol response), OT (if detectable), but since especially the latter is rather meaningful challenge based, we will additionally measure OT/T/C before and after the MR task (same day as baseline measures), hence before and after the perception of a neutral and aggressive film sequence (three times), to be able to relate baseline with challenge based measures. To assess saliva samples for the analysis, participants will be asked to passively drool saliva into a polypropylene sampling tube for one minute. Saliva samples will be frozen at least 20 degrees below zero (Celsius). For cortisol, they will be analyzed at the Institute of Medical Psychology’s biochemical lab (University Hospital Heidelberg, Heidelberg, Germany) using standard commercially available enzyme-linked immunosorbent assays (ELISA) according to the manufacturer’s protocol. For the analyses of oxytocin concentrations saliva samples will be analyzed following the protocol of oxytocin enzyme-linked immunosorbent assay from Enzo Life Sciences (ELISA; ENZO Life Sciences, Switzerland), while for assessments of the steroid hormones (testosterone, progesterone and estradiol), the chemiluminescent assay from IBL International (CLIA; IBL International, Germany) will be used.

**Environmental factors:** A number of assessments will be used to also explore actual and past life events and stressors as indicated above: Childhood Trauma Questionnaire (CTQ) [76] as well as a semi-standardized medical history in children and youth. Sociodemographic and treatment information as well as clinical history of the patients will be assessed at baseline and follow-up assessment points (comparable to the DZPG core dataset).

**Brain measures:** The MRI protocol for children and adults will involve state-of-the-art magnetic resonance imaging (MRI, 3 Tesla) of the brain enabling identical imaging procedures for the assessments across acquisition sites.

Based on shared standard operation procedures the sites will acquire the following measures:

MR-anatomy (12.32 min); fMRI resting state measurement (8 min); fMRI with concurrent aggression perception task (violent movie scenes, 24 min) in adults, as well as age-appropriate aggression footage in children and adolescents; multidirectional diffusion imaging (7.25 min).

MR-anatomy (6.38 min): 3D T1-weighted MPRAGE sequence.

MR-anatomy for myelinization (5,54 min): 3D T2-weighted SPACE sequence in the same measurement space as T1-anatomy.

fMRI resting-state (8 min): Subjects will be instructed to rest with their eyes open for 8 minutes.

fMRI with naturalistic viewing paradigm for aggression perception (12 min each): Participants will watch violent movie scenes (from [74]) during the fMRI measurements. Five highly violent movie clips will be used. They show the use of physical force to inflict harm to another person and come from the following movies: Man on Fire (2004) – Platoon (1986) – Precious (2009) – Leon: The Professional (1994) –and Saving Private Ryan (1998). The total duration of the clips is around 12 minutes, with each clip lasting 2–3 minutes. Five non-violent clips matched with the violent clips on duration and high level of action, are used as control and were rated as non-violent (*M* = 1.40, SD = 0.29). These clips come from the following movies: Twister (1996) – Crash (2004) – Speed (1994) – Castaway (2000). In case of children (<16 years), they will watch age adequate videoclips of violent (physical, verbal, peer and parental) conflicts (2–3 min, in total 12 minutes) taken from a German children’s TV serie (Pfefferkörner) which have been validated by youth and approved by the Ethical committee of Aachen University hospital for use in children and adolescents. After measurements, subjects will be asked about their state and experience of the condition.

Multidirectional diffusion-weighted imaging (7.25 min).

**Genetics**: DNA from blood or saliva will be obtained at baseline and will be analyzed on a genome-wide level. We will form a composite aggression score from the phenotypic measures obtained via questionnaires and EMA (see above) and then construct an “aggression polygenic risk score” (AGG-PRS) from our sample. The rG (genetic risk) of AGG-PRS with disease PRS from the PG Consortium will be calculated, but also to other phenotypic PRS e.g. from UK Biobank to uncover the genetic architecture of AMD and its unique respectively shared genetic information with psychiatric disorders as such. Correlational analyses with functional and structural brain measures, hormone levels, and trajectories over time will also be performed. Additionally, blood or saliva samples will be collected annually for future epigenetic analyses. Samples will initially be stored and analyzed at a later stage using state-of-the-art methodologies.

**Microbiome:** In order to examine the individual composition of intestinal bacteria and their connection with behavior (gut microbiome), we want to examine a stool sample from the participants. In addition, two questionnaires (the Bristol Stool Scale and the Nutritional Questionnaire) will be administered to gather complementary information and provide a more comprehensive understanding of how diet and digestive health may influence the gut microbiome. The stool sample can be collected at the study site or at home following detailed instructions. Samples will be collected annually.

Stool samples will undergo metagenomic analysis to characterize and compare the composition and functional properties of the microbiome of the respective participants. Microbial genomic DNA will be isolated using commercially available kits. For metagenomic analyses, DNA extracts from the biological samples will be sequenced on a NovaSeq platform using 2 × 150 bp paired-end reads.

**Power calculation: **The sample sizes of our study design were determined based on power calculations according to classical test theory (CTT), which were conducted using the G*Power 3 software [77]. We initially estimated the necessary sample size as a function of (i) the significance level alpha (i.e., nominal type I error), (ii) the desired statistical power 1-beta, and (iii) the to-be-detected population effect size. For all analyses examining significant between-group differences via t-test for independent samples, it can be shown that a total sample size of *N* = 352 (*N* = 176 individuals per group) is required to detect a small to medium effect of 0.3 with a power of 0.8 (alpha = 0.05, two-tailed). For all correlational relationships, a sample size of *N* = 82 is required to detect a small effect size of 0.3 with a power of 0.8 (alpha = 0.05, two-tailed). Accordingly, after stratifying individuals based on distinct aggression-related biosignatures (Hypothesis 1), our total sample of *N* = 1,100 participants with heterogeneous mental health conditions provides sufficient statistical power to support comparative analyses across up to six putative subgroups defined by psychometrical profiles (see Hypothesis 2). Notably, the statistical power of the cluster-analytic approach used to establish these subgroups is mainly driven by the expected effect sizes separating the groups. More specifically, simulation studies indicate that when individuals are subdivided on the basis of 50 features (e.g., genetic, hormonal, and neural correlates of aggression), assuming small effects (Cohen’s d = 0.3) for 30 features, medium effects (d = 0.5) for 15 features, and large effects (d = 0.8) for five features, a total sample size of *N* = 160 is already sufficient to identify up to four equally sized clusters with a statistical power of 0.88 using, for example, k-means clustering [78]. Accordingly, our planned sample of *N* = 1,100 seems more than adequately powered to delineate and statistically compare up to six putative aggression-related subtypes or biosignatures.

Power estimation for predictive biomarker models (Hypotheses 3–4) is intrinsically challenging and typically relies on simulation-based approaches using pilot data. This is further complicated in machine-learning (ML) settings by high-dimensional feature spaces and potential class imbalance. Recent methods, however, allow estimation of the sample size required to achieve predefined levels of predictive performance. For example, the R package planningML can be used to model expected area under the receiver operating characteristic curve (AUC) as a function of sample size, enabling identification of the minimum cohort size needed to reach, for instance, 80% accuracy. These approaches will be implemented once pilot data becomes available. Simulation studies further indicate that ML performance is primarily driven by the average and overall effect sizes of the predictor set [79]. In low-effect regimes (d ≈ 0.1), predictive accuracy typically plateaus around 60%, even in large samples (*N* > 1,000), whereas moderate effects (d ≈ 0.5) can yield accuracy approaching 80%. Accordingly, in our cohort of *N* = 1,100, we will prioritise features with effect sizes exceeding 0.5 prior to model fitting, a strategy expected to improve both predictive accuracy and model robustness.

To address Hypothesis 1 (i.e., the identification of putative neurobiological aggressor subtypes), we aim to stratify individuals with mental health conditions based on neuroimaging-derived phenotypes (sMRI, DTI, rsfMRI), hormonal assessments, and genetic profiles. To this aim, we will employ a variety of supervised and data-driven (i.e., unsupervised) clustering techniques such as k-means clustering, hierarchical clustering, and/or Gaussian Mixture Modelling. For unsupervised clustering, the optimal number of clusters will be identified in a data-driven fashion by evaluating solutions for different numbers of clusters across multiple validity indices (frey, mcclain, cindex, silhouette, and dunn index). The robustness of the clustering solution will be established via hold-out methods, where the clustering algorithm is initially applied to a randomly drawn subset of the sample (e.g., 75%) and subsequently tested based on the remaining datasets (e.g., 25%). Moreover, using a variety of conventional statistical (GLM-type) approaches, we aim to validate the biologically defined clusters against clinical and behavioral measures of aggression (see Hypothesis 2), and establish if subgroups converge onto distinct behavioral or clinical aggressor phenotypes. In particular, we will compare subgroups in terms of the profile and severity of aggressive symptoms and traits and relate these to the CS and NVS via correlation and regression analyses. As soon as the longitudinal Q1 data becomes available, these analyses will be repeated to establish whether subtypes are stable (or variable) across development, and how subgroup membership relates to diagnostic categories and sex differences (Hypothesis 3 and 4). A linear mixed effects (LME) model will be used to determine if subgroups also differ in terms of their neurodevelopmental and clinical outcome (Hypothesis 5), and to select features that are most predictive of developmental trajectories. To control the risk of type I errors arising from multiple statistical testing, *p*-values will be adjusted for using the False Discovery Rate (FDR) estimation. In the second funding period, measures exhibiting the largest effect sizes in the comparative analyses conducted during the first period will be prioritised and integrated into a machine-learning (ML) framework to enable individual-level prediction of transdiagnostic CS- and NVS-related aggressor phenotypes and biosignatures, as well as personalised forecasts of long-term developmental trajectories and clinical outcomes.

## Discussion

In this naturalistic, one-arm study, randomization and blinding does not apply. Comparison groups will be constituted based on their quantified aggression level. Study personnel (clinician scientist at each site) will be trained in the assessment procedures that are standardized (clinical interviews), computerized (Questionnaires, JTrack) and accompanied by standard operating procedures (SOP). The computerization allows for immediate access to standardized data files, reduces data cleaning efforts and enhances objectivity and quality. Repeated assessments every 12-months will be carried out by clinically trained assessors. Quality control will be assured by annual supervision of procedures by the quality control group at each site. Supervision of recruitment will be performed by monthly meetings of applicants. Thus, a high standard of quality control and comparable recruitment and assessment procedures are guaranteed. An in-depth documentation as well as file documentation will be performed. This will be conducted at each site and assessment point and contains baseline sociodemographic, clinical, and therapeutic (psychotherapy as well as medication) information and dropout documentation. Clinical project managers are responsible for the timely and complete documentation.

In case attrition is high, additional efforts in recontacting subjects and learning about their reasons to quit will be made; corresponding adaptations to the reimbursement schedule and/or the contacting procedures will be made (mitigation plan 1). In case one site underperforms, we will shift resources to the well-recruiting site and participants (mitigation plan 2). In case of drop-outs, we will recruit additional patients to maintain the envisaged sample sizes.

## Data Availability

No datasets were generated or analysed during the current study.

## Abbreviations

ABCL: Adult Behavior Checklist
ACE: Adverse Childhood Experiences
ADHD: Attention Deficit/Hyperactivity Disorder
AGG-PRS: Aggression Polygenic Risk Score
AMD: Aggression in Mental Disorders
AMDP: Alternative Model for Personality Disorders
ARS: Anger Rumination Scale
ASD: Autism Spectrum Disorder
ASPD: Antisocial Personality Disorder
ASR: Adult Self Report
BIS: Barratt Impulsiveness Scale
BIS/BAS: Behavioral Inhibition Scale
BPD: Borderline Personality Disorder
C: Cortisol
CBCL-R: Child Behavior Checklist Revised
CD: Conduct Disorder
CGAS: Children’s Global Assessment Scale
CS: Cognitive System
CTT: Classical Test Theory
CTQ: Childhood Trauma Questionnaire
DB: Digital Biomarkers
DERS: Difficulties in Emotion Regulation Scale
DS: Digit Span
DSM: Diagnostic and Statistical Manual of Mental Disorders
DTI: Diffusion Tensor Imaging
E: estrogen
ELISA: enzyme-linked immunosorbent assays
EMA: Ecological Momentary Assessment
ERQ: Emotion Regulation Questionnaire
fMRI: Functional Magnetic Resonance Imaging
GDPR: General Data Protection Regulation
GWAS: Genome-Wide Association Studies
ICD: International Classification of Diseases
ICU: Inventory of Callous-un-emotional traits
IPDE: International Personality Disorder Examination
LHA: Life History of Aggression Scale
LPFS: Level of Personality Functioning Scale
MDS: Minimum Data Set
MHF: Medical History Form
MINI-DIPS: Mini International Neuropsychiatric Interview
ML: Machine Learning
MR: Magnetic Resonance
NIMH: National Institute of Mental Health
NVS: Negative Valence System
O: oxytocin
OAS-M: Modified Overt Aggression Scale
OBQ-R: Olweus Bullying Questionnaire – Revised
PAG: Periaqueductal Grey
PDS: Pubertal Development Scale
PR: Progesterone
PRS: Polygenic Risk Scores
QDF: Questionnaire of Daily Frustrations
RCADS: Revised Child Anxiety and Depression Scale
RDoC: Research Domain Criteria
RPQ: Reactive Proactive Aggression Questionnaire
SOFAS: Social and Occupational Functioning Assessment Scale
SOP: standard operating procedure
SSQ: Social Support Questionnaire
STAXI: State-Trait-Anger-Expression-Inventory
T: testosterone
TSS: Threat Sensitivity Scale
V: Vocabulary
WAIS: Wechsler Adult Intelligence Scale
WISC: Wechsler Intelligence Scale for Children
YSR: Youth Self Report
